# Quality control monitoring of euploid frozen embryo transfers

**DOI:** 10.1007/s00404-026-08474-4

**Published:** 2026-06-16

**Authors:** Michael S. Awadalla

**Affiliations:** Institute for Reproductive Health, Cincinnati, OH USA

**Keywords:** Euploid embryo, Frozen embryo transfer, Embryo transfer protocol, Letrozole, Modified natural cycle

## Abstract

**Purpose:**

The goal of this study is to determine factors associated with fetal heartbeat and live birth rate for euploid frozen embryo transfers.

**Methods:**

This is a retrospective cohort study of IVF patients at a single private practice in Cincinnati, Ohio undergoing single frozen euploid embryo transfer from 1/1/2023 to 7/31/2025. Variables assessed are embryo transfer protocol, genetic testing laboratory, physician who performed the embryo transfer, and embryologist. The main outcomes of interest are fetal heartbeat and live birth rate per single euploid frozen embryo transfer.

**Results:**

Fetal heartbeat rate was significantly associated with the frozen embryo transfer protocol (*p* < 0.01). Modified natural FET protocols without oral medication (such as letrozole) had the highest fetal heartbeat rate of 66% compared to the average fetal heartbeat rate of 52%. Fetal heartbeat rate was also associated with the genetic testing laboratory (*p* < 0.01) and the day of embryo biopsy (*p* < 0.01). Fetal heartbeat was not associated with any of the other modifiable measures evaluated including the embryologist performing the biopsy or the physician performing the embryo transfer.

**Conclusion:**

At our center, the highest ongoing pregnancy rate per euploid embryo is seen with modified natural FET protocols without oral medication (such as letrozole).

## What does this study adds to the clinical work

**Table:** 

This study gives detail on the benefits of the corpus luteum in uterine receptivity and luteal phase support. The results provide new and clinically relevant insights into frozen embryo transfer protocols including the negative impact of letrozole on endometrial receptivity in modified natural frozen embryo transfer protocols.	

## Introduction

Until 2015, most embryo transfers were non-PGT-tested embryos where the expected live birth rate could range anywhere from 1 to 50% which made evaluation of FET protocol performance challenging [1]. Evaluation of euploid embryo transfers removes most embryo quality variability but introduces variability in the skill of the biopsy embryologist. One study found that it may take up to 3 years and 3000 embryo biopsies to obtain proficiency at embryo biopsy [2]. Some studies do not include details for modified natural embryo transfer cycles such as if letrozole or Clomid were used. Some systematic reviews suggest that total natural FETs and modified natural FETs are associated with higher live birth rates than hormone replacement FET protocols [3]. Single center data is needed to evaluate this further and assess the effect size.

There is biological plausibility both for and against using letrozole in frozen embryo transfer cycles. Although letrozole may increase the number of corpora lutea which may increase progesterone production, letrozole may worsen endometrial preparation by lowering the estradiol level.

Typically, the term “total natural FET” refers to ultrasound and blood monitoring only with no administration of any medications. The term “modified natural FET” describes embryo transfer after ovulation in combination with the use of any medications during the FET cycle such as FSH, letrozole, Clomid, HCG trigger, and/or luteal phase support with progesterone.

The aim of this study is to determine which factors are associated with ongoing pregnancy and live birth rates after euploid embryo transfer. Polycystic Ovarian Syndrome (PCOS) is an important consideration because it is not always possible to determine which patients have PCOS from record review. Many patients with PCOS do modified natural FET cycles using ovarian stimulation with letrozole or follicle-stimulating hormone (FSH). Patients without PCOS typically do modified natural FET cycles with no ovarian stimulation or with letrozole. The impact of the genetic testing company, embryologist, and physician on embryo transfer success is also evaluated in detail. The corresponding author can be contacted at msawadalla@irhfertility.com.

## Methods

### Study population

This retrospective cohort study included 844 single euploid embryo transfers from 1/1/2023 to 7/31/2025 for patients undergoing IVF at the Institute for Reproductive Health in Cincinnati, OH. This is a data-limited study. The study was approved by the Christ Hospital Institutional Review Board (#25–030). A full waiver of informed consent was obtained by the IRB and chart review was done with HIPPA compliance.

Transfers involving gestational carriers, donor oocytes, and reciprocal IVF were included.

For gestational carrier and donor oocyte embryo transfers, the age of the women at the time of oocyte retrieval was used in the analysis. Nine hundred thirty-four embryo transfers met the inclusion criteria. There were a total of 90 embryo transfers excluded for the use of embryos vitrified at other centers (*n* = 41); embryos that were thawed, biopsied, and refrozen (*n* = 28); repeat biopsy (*n* = 7); transfer of a segmental aneuploid embryo (*n* = 2); and transfer of a “no result” or “inconclusive” embryo (*n* = 12). There were no mosaic embryos transferred. Eight hundred forty-four single euploid embryo transfers were used in the final analysis.

The average age at oocyte retrieval was 34 years (SD 4 years). When the age of the oocyte donor was not known, the age of 27 years was used. The mean weight was 165 lbs (SD 41 lbs), height 65 inches (SD 3 inches), and BMI 28 kg/m^2^ (SD 7 kg/m^2^). Fetal heartbeat rate was not associated with BMI and BMI was not adjusted for in the analysis. Of the 844 euploid embryo transfers, 368 (44%) were day 5, 419 (50%) were day 6, and 57 (7%) were day 7. ICSI was used for 82% of cases and conventional insemination for 18%. The genetic testing laboratories used were Cooper Genomics (*n* = 99, 12%), Genomic Prediction (*n* = 292, 35%), Igenomix (*n* = 4), Ovation LifeView (*n* = 304, 36%), Ovation with next generation sequencing (NGS, *n* = 130, 15%), and Progenesis (*n* = 15, 2%). Ovation used the LifeView technology in-house which was licensed from Genomic Prediction and used in-house data interpretation. Ovation performed NGS in-house. Genomic Prediction and Ovation LifeView used a SNP array for PGT-A testing, while the others used NGS.

Fetal heartbeat was measured 4 weeks after frozen embryo transfer (at 6 weeks 5 days gestation) by transvaginal ultrasound performed by a physician specializing in reproductive endocrinology and infertility or a nurse practitioner with over 2 years of experience with transvaginal ultrasound.

### IVF and laboratory methods

Ovarian stimulation was performed with recombinant human (FSH) 75–300 IU SC daily (Follistim: Merck, Kenilworth, NJ; or Gonal-f: EMD Serono, Rockland, MD) and/or human menopausal gonadotropins (hMG) 75–150 IU SC daily (Menopur: Ferring Pharmaceuticals, Parsippany, NJ). Our most common dosing was 225 IU of FSH SC and 75 IU of hMG SC starting on cycle day 3. Approximately 50% of patients did 9 days of stimulation, 40% did 10 days, and 10% did more than 10 days.

Pituitary suppression was achieved with gonadotropin-releasing hormone (GnRH) antagonist, GnRH agonist suppression, or GnRH flare suppression protocols. Approximately 95% of stimulation cycles used GnRH antagonist. In antagonist cycles, the GnRH antagonist (0.25 mg SC ganirelix acetate daily or 0.25 mg SC cetrorelix acetate daily) was started when the lead follicle reached 12–14 mm in diameter, typically on day 6 of stimulation. When deemed appropriate, patients received a trigger medication for oocyte maturation and the GnRH antagonist was discontinued. Our most common trigger dose was Ovidrel 250 µg (EMD Serono) if the body weight was 200 lbs or less and Ovidrel 500 µg for over 200 lbs. Sometimes a co-trigger with the addition of leuprolide 4 mg SC was administered. If hyperstimulation was a concern, a leuprolide-only trigger was often used or a leuprolide 4 mg SC with half of an Ovidrel (125 µg) SC was used. Follicular aspiration was performed 35.5 h after the trigger administration. Doxycycline 100 mg PO once was administered on the day of oocyte retrieval.

Oocyte cumulus complexes were identified in the follicular fluid with an Olympus SZX16 microscope and transferred to oocyte collection dishes in Modified HTF with gentamicin (Irvine Scientific, Santa Ana, CA). Oocytes were placed in Global for Fertilization, or GFF (LifeGlobal, Guilford, CT) before insemination or ICSI. IVF insemination was carried out 4–6 h after the retrieval with each droplet being inseminated with 100,000 motile sperm. ICSI was performed on mature MII oocytes after removal of cumulus coronal cells. Removal of cumulus coronal cells was performed by exposure to hyaluronidase for 60 s while pipetting with a glass Pasteur pipet (Origio, Turnbull, CT) followed by transfer to GFF and pipetting with 200 μm, 170 μm, and 150 μm diameter pipettes. ICSI was performed with a Nikon Eclipse Ti2 microscope (Nikon Metrology, Tokyo, Japan) with a heated stage (Tokai Hit, Shizuoka, Japan) and a Narishige micromanipulator (Narishige, Tokyo, Japan). At 16–18 h post insemination or ICSI, a fertilization check was performed.

Embryos were cultured in 75–100 μL drops of Continuous Single Culture Complete with Gentamicin and Human Serum Albumin (HSA) media (CSCM-NXC, Irvine Scientific, Santa Ana, CA). Embryos were cultured in BIRR ART 4 + 8-well Dishes (BIRR BioSciences, Netherlands) overlaid with Ovoil Heavy (Vitrolife, Englewood, CO) for 7 days. The incubator conditions were set to a temperature of 37.0 °C, 5% O2, 6.3% CO2, and 88.7% N2. The % CO2 was titrated to maintain the culture media pH at 7.2–7.4 with a preferred pH range of 7.25–7.35. The embryos were cultured in a Miri tabletop incubator (ESCO Medical, Changi, Singapore). Until October 2024, the laboratory performed laser-assisted hatching on day 3 for all PGT cases to assist with biopsy at the blastocyst stage. In October 2024, the laboratory discontinued hatching on embryo day 3. Embryos were biopsied when they reached an expansion stage of 4 (fully expanded blastocyst with thinning of the zona) [4].

### Frozen embryo transfer protocols

For hormone replacement therapy (HRT) FETs, estradiol 2 mg PO TID was started on cycle day 3 and a transvaginal ultrasound (TVUS) was performed on cycle day 12–16. If the endometrium was 7.0 mm or greater and trilaminar in appearance, progesterone was started and the embryo was transferred on the 6th day of progesterone. Our most common progesterone protocols were Prometrium 200 mg PV TID, Endometrin 100 mg PV TID, Prometrium 200 mg PV TID with progesterone in oil (PIO) 50 mg IM every 3rd day (both started on day 1 of progesterone), Endometrin 100 mg PV TID with PIO 50 mg IM every 3rd day, and PIO 100 mg IM every day. Sometimes vaginal progesterone four times a day was used. Since progesterone started in the morning and we perform embryo transfers mostly from 9 AM to noon, this equated to approximately 120 h from progesterone start to embryo transfer. Patients are instructed to discontinue all estrogen and progesterone at 10 to 12 weeks gestation per physician preference.

For modified natural embryo transfers, we typically performed a TVUS on cycle day 12 and checked serum levels of estradiol, progesterone, and LH. Our typical dose of letrozole was 5 mg PO daily on cycle days 3–7. Our typical gonadotropin dose was 75 units of FSH (Gonal-f or Follistim) SC every other day starting cycle day 3. When the lead follicle was 16–23 mm in mean diameter, 250 µg of recombinant choriogonadotropin alfa (Ovidrel, approximately 6500 IU of hCG) was administered at 6–10 PM. Vaginal micronized progesterone (most commonly Prometrium 200 mg PV daily) was started 4 days after the hCG trigger. The embryo transfer was scheduled 7 days after the trigger. For example, if the hCG trigger was on Tuesday July 1st, the transfer would be on Tuesday July 8th. The embryo transfer could be moved one day sooner for an elevated LH on the day of trigger per physician preference. This equates to approximately 156 h from trigger to embryo transfer.

All 844 embryo transfers were categorized into one of five general FET protocol groupings: (1) vaginal progesterone TID to 4x/day, (2) vaginal progesterone TID and PIO IM every 3 days, (3) modified natural with letrozole or tamoxifen, (4) modified natural without letrozole or tamoxifen, and (5) 100 mg PIO IM daily. The modified natural group with oral medication included 9 cycles where tamoxifen was used and 45 where letrozole was used for simulation for a total of 54 embryo transfers in this group. The modified natural without letrozole group included 7 euploid embryo transfers after oocyte retrieval, 1 total natural FET, 48 FETs with gonadotropin stimulation using mostly FSH, and 4 participants in the Progress Study (Ovidrel trigger followed by subcutaneous or vaginal progesterone for luteal phase support) [5].

### Data analysis and statistical methods

We evaluated seven primary modifiable variables for association with fetal heartbeat: FET protocol group, genetic testing company that performed the PGT-A, biopsy embryologist, vitrification embryologist, thaw embryologist, embryologist that performed the transfer, and physician that performed the transfer.

Fisher’s exact tests were used to determine if variables were associated with fetal heartbeat. To adjust for evaluation of seven variables, a p value of less than 0.01 was considered statistically significant. Post hoc analyses were performed using a Bonferroni correction for calculating adjusted *p* values. 95% confidence intervals were calculated using the Clopper–Pearson exact method. Logistic regression was used to assess the relationship between age at oocyte retrieval and fetal heartbeat. Stata version 16.1 was used for statistical analysis.

Several secondary exploratory analyses were performed. These included evaluating the day of embryo biopsy, age at oocyte retrieval, ICSI vs conventional insemination, and the impact of FET protocol (ovulatory vs non-ovulatory) on the rate of fetal heartbeat based on initial hCG. A subgroup analysis was performed comparing modified natural FETs without ovarian stimulation, modified natural with oral medication for ovarian stimulation, and gonadotropin-stimulated modified natural FETs. We also evaluated Endometrin 100 mg PV vs Prometrium 200 mg PV in HRT FETs. For ad hoc exploratory evaluation of modified natural cycles, a *p* value of less than 0.05 with biological plausibility was considered significant. A limited dataset is available through Mendeley Data [6]. Potential confounding by day of embryo biopsy and age at retrieval was assessed by evaluating average embryo day and average age at retrieval among groups.

## Results

**Fig. 1:**
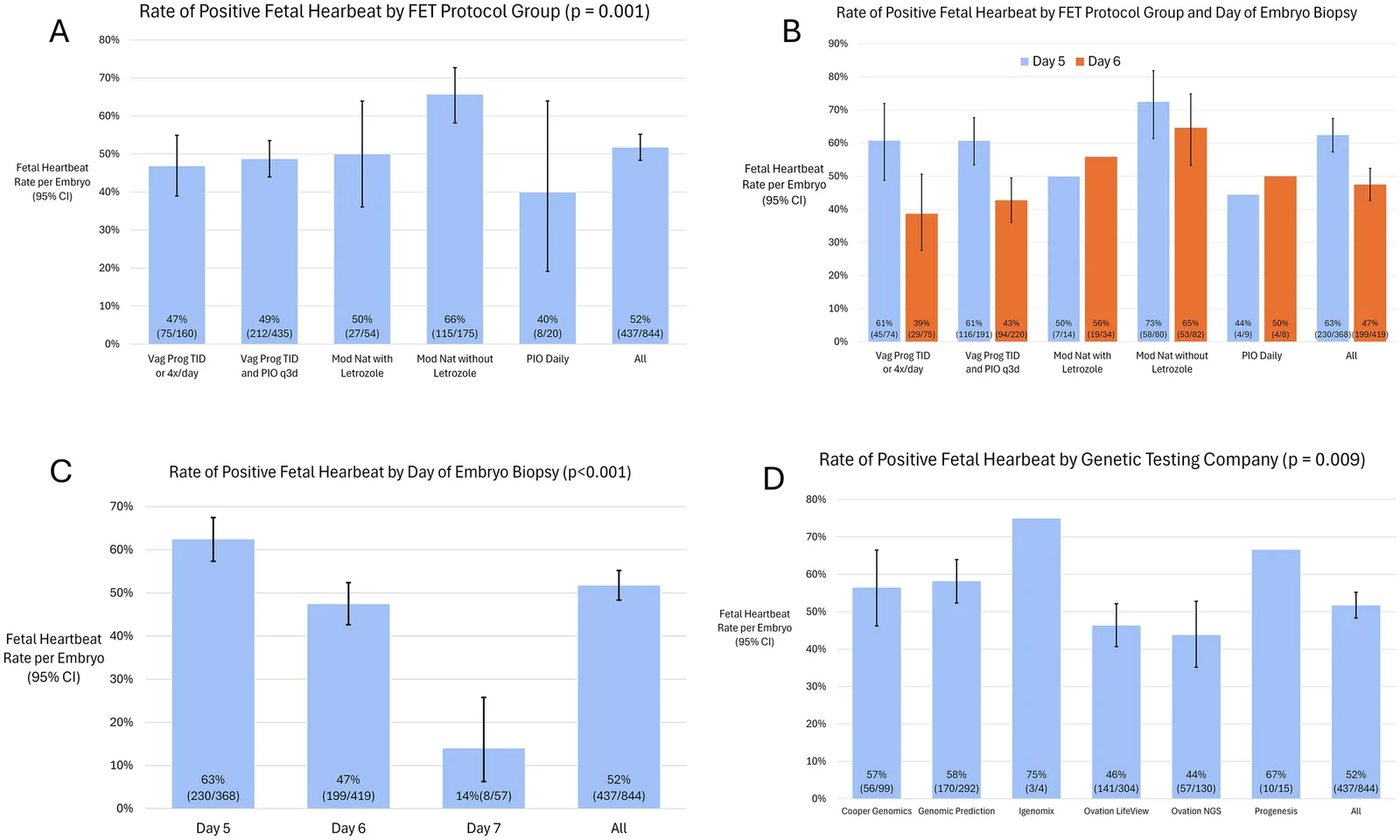
Variables associated with fetal heartbeat

The only modifiable factors that were significantly associated with fetal heartbeat rate were FET protocol and PGT-A laboratory (Fig. 1). Day of embryo biopsy and age at oocyte retrieval were non-modifiable variables associated with fetal heartbeat. There were 2 cases of MonoDi twins out of 437 pregnancies that progressed to have a heartbeat. In the analysis, these were counted the same as transfers with 1 heartbeat.

Modified natural FETs without letrozole performed significantly better than vaginal progesterone-only HRT FETs (adjusted *p* value of 0.005) and vaginal progesterone TID with PIO every 3 days (adjusted *p* value of 0.001). This association with a similar effect size was also seen in a subgroup analysis broken down for day 5 and day 6 embryos (Fig. 1A, B). There were no significant differences in average day of embryo biopsy or average age at oocyte retrieval for any of the FET protocol groups. Data for embryos by day of biopsy are shown in Fig. 1C.

PGT-A genetic testing company was significantly associated with fetal heartbeat as shown in Fig. 1D (*p* = 0.009). There were no significant differences in the average day of embryo biopsy or the average age at oocyte retrieval for any of the genetic testing companies.

For day 5 and day 6 euploid embryos, the fetal heartbeat rate was 54% for embryos fertilized by ICSI and 56% for conventional insemination (*p* = 0.65). The association between age and fetal heartbeat was not statistically significant. The best fit fetal heartbeat rate based on age at oocyte retrieval varied from 56% at age 25 years to 47% at age 45 years. The best fit relationship between age at oocyte retrieval in years and fetal heartbeat rate was 0.6749–0.0046 *Age. This result agrees with a recent meta-analysis [7].

**Fig. 2:**
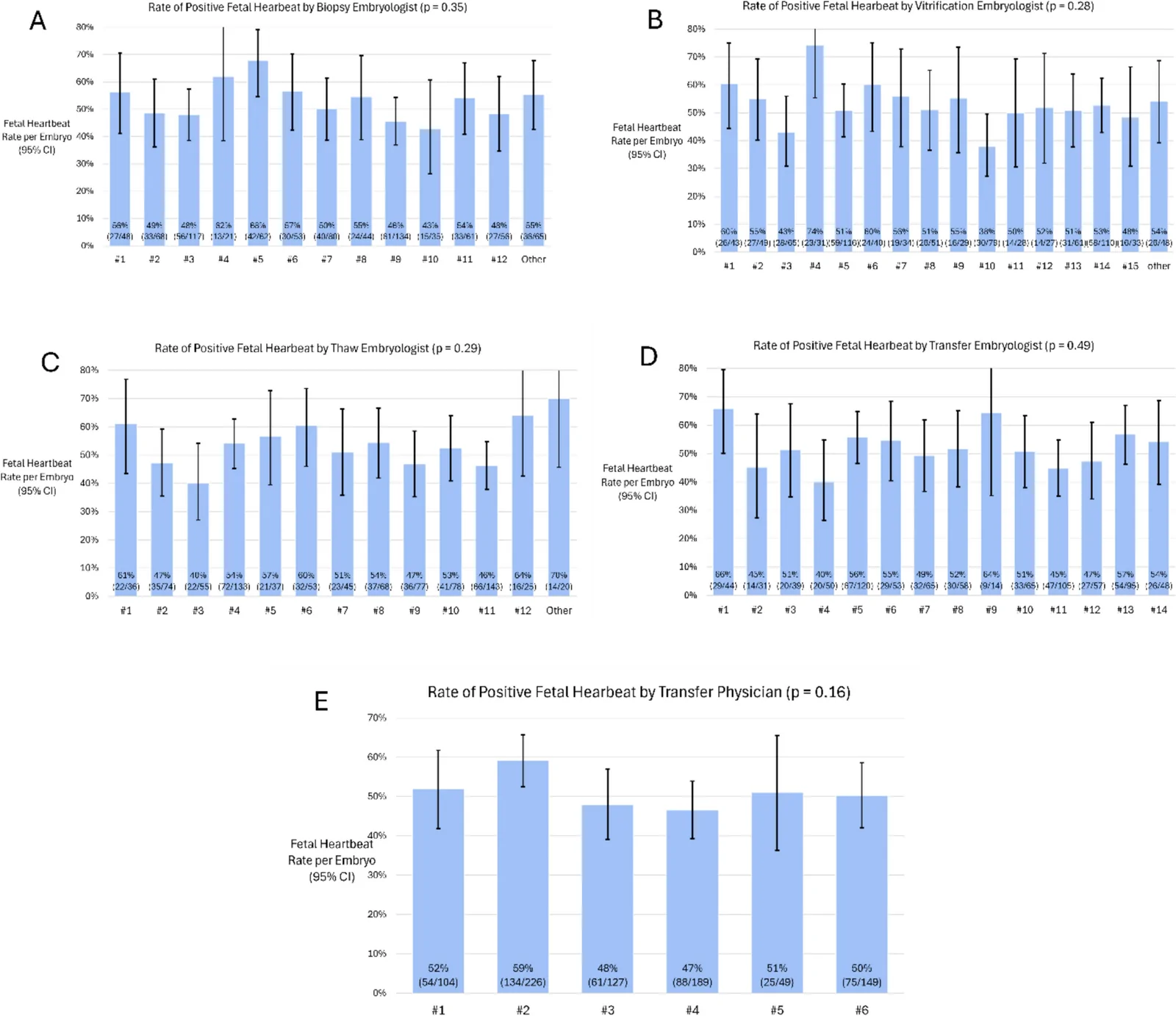
Variables not associated with fetal heartbeat

**Fig. 3:**
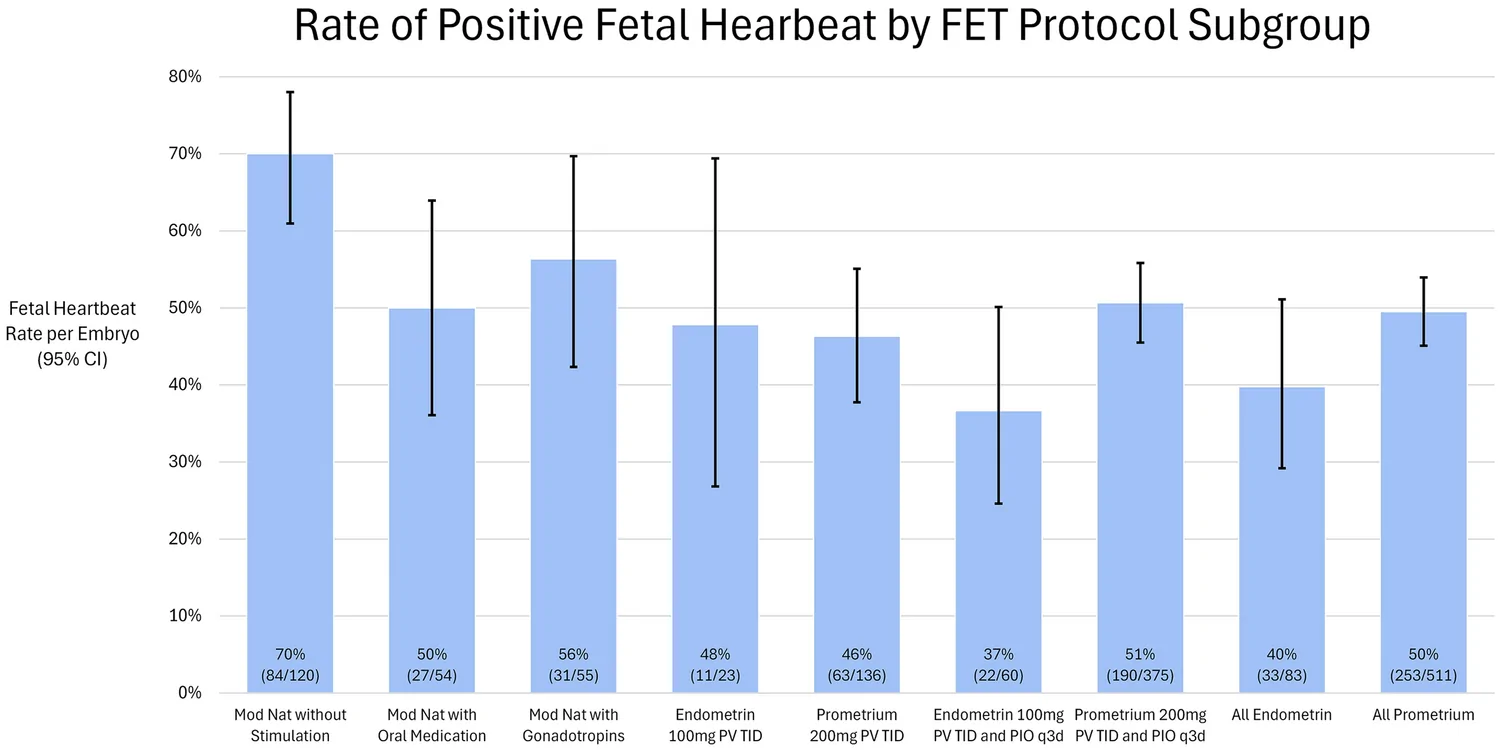
Subgroup analysis of FET protocols

Embryologist and physician who performed the embryo transfer were not associated with fetal heartbeat rate as shown in Fig. 2. Our subgroup analysis showed that modified natural cycles without stimulation had a higher fetal heartbeat rate of 70% compared to 50% for modified natural cycles with oral medication (Fig. 3, *p* = 0.01). This may not be entirely modifiable as anovulatory patients with PCOS require ovarian stimulation for ovulation. However, this suggests that gonadotropins may be a better option for ovarian stimulation than letrozole.

**Fig. 4:**
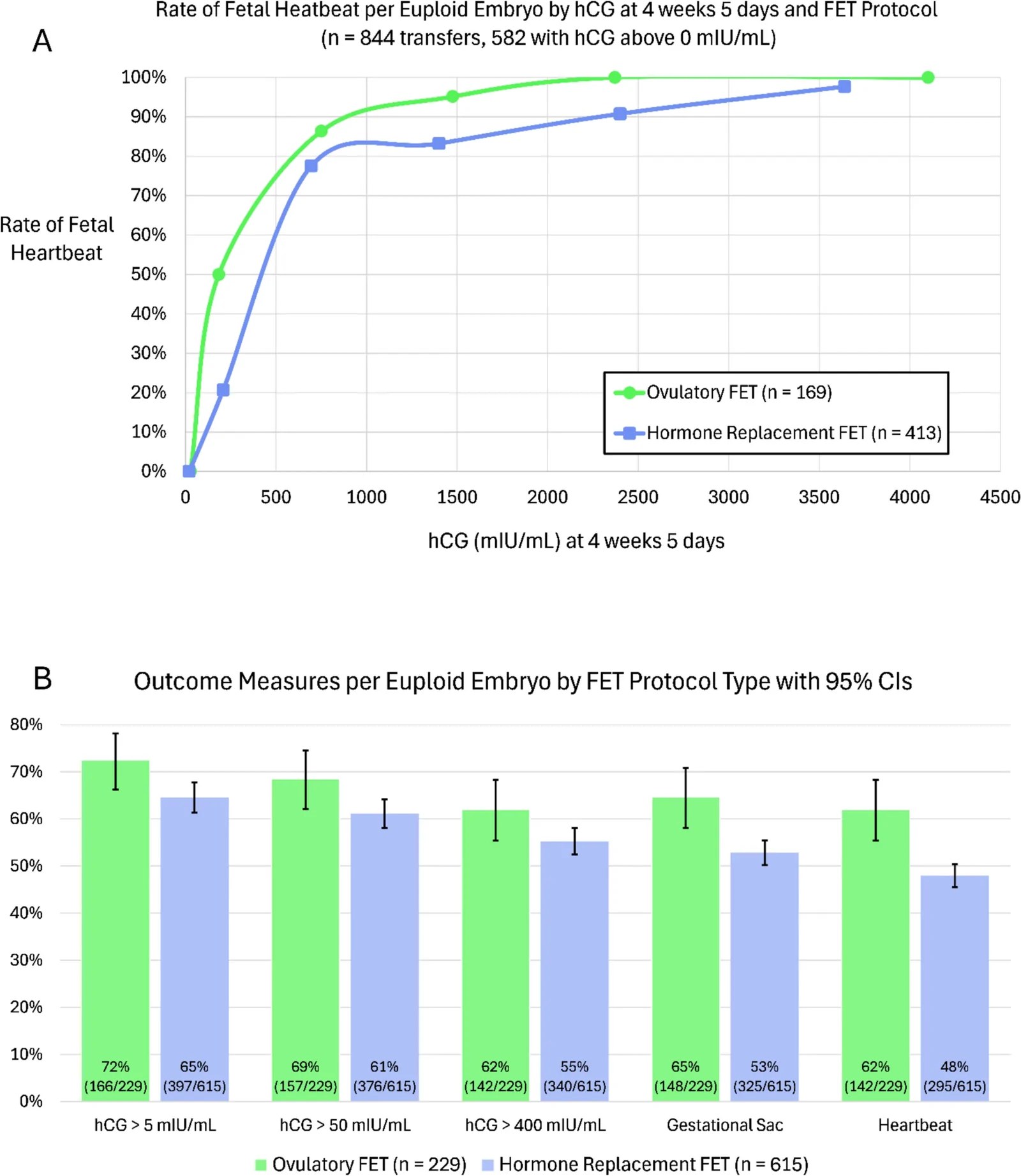
Transfer outcomes by FET protocol type

When comparing all transfers after an ovulation (natural FETs, modified natural FETs, and transfer of a euploid embryo after an oocyte retrieval), the rate of hCG above 5mIU/mL was 72% compared to 65% for HRT cycles. The fetal heartbeat rate was 62% for ovulatory FETs compared to 48% for non-ovulatory FETs. High initial hCG was associated with a higher rate of fetal heartbeat (Fig. 4A, B).

Full live birth data were available for the first 357 single euploid embryo transfers which resulted in 171 ongoing pregnancies and 160 live births. When a heartbeat was detected, a live birth followed 94% of the time. For ovulatory FET cycles, a live birth was seen in 26 of 27 cases where a fetal heartbeat was detected (96%). For HRT FET cycles, a live birth was seen in 134 of 144 cases where a fetal heartbeat was detected (93%). Of the 11 instances where a fetal heartbeat was detected and no live birth occurred, 8 of these were spontaneous abortions at 6 to 9 weeks. One was a 20-week loss attributed to an umbilical cord accident. Another was a stillbirth at 22 weeks attributed to cervical incompetence. The other was a monochorionic diamniotic twin pregnancy loss due to unexplained IUFD at 14 weeks. Of the 163 pregnancies that were ongoing at 10 weeks, 160 resulted in a live birth (98%).

## Discussion

These findings suggest that modified natural FET cycles without oral medications (letrozole, clomiphene, or tamoxifen) are likely to achieve the highest rates of ongoing pregnancy and live birth. Low-dose gonadotropin stimulation (such as 75 IU SC every other day) appears to be a better option than letrozole to induce ovulation in patients with PCOS. While our clinic typically uses an hCG trigger and vaginal progesterone, some data suggest they do not impact ongoing pregnancy rates in modified natural FETs [8]. The results of this retrospective cohort study are limited by confounding by indication and the results are hypothesis generating rather than definitive. These data can be used for power analysis for future prospective randomized controlled studies. Another limitation is that fetal heartbeat, rather than live birth, is used as the main outcome measure as many of the pregnancies were still ongoing at the time of the data compilation.

When comparing all transfers after an ovulation (natural FETs, modified natural FETs, and transfer of a euploid embryo after an oocyte retrieval), the rate of HCG above 5 mIU/mL was 72% compared to 65% for HRT cycles as shown in Fig. 4B. This likely represents approximately 7% higher endometrial receptivity for FETs after ovulation. The fetal heartbeat rate was 62% for ovulatory FETs compared to 48% for non-ovulatory FETs. The drop-off from positive hCG to fetal heartbeat was an absolute value of 10% for ovulatory FETs (72 to 62%) and 17% for HRT FETs (65 to 48%). This likely represents a 7% higher absolute advantage of luteal support with embryo transfer after ovulation. Thus, of the 14% higher rate of fetal heartbeat per euploid embryo for transfers after ovulation (62% compared to 48%), half can be attributed to better endometrial receptivity and half to better luteal phase support. Several centers on the West Coast of the United States seem to have high live birth rates with euploid embryos using a combination of 100 mg PIO IM daily with vaginal progesterone twice a day. It is uncertain how these high-dose progesterone protocols compare to modified natural FETs.

Although this data suggests that the corpus luteum is the most important factor to increasing ongoing pregnancy and live birth rates for FETs, a 2017 Cochrane review found no benefit to modified natural FET cycles compared to HRT FETs [9]. For hormone replacement FETs, there was no benefit in adding 50 mg of IM progesterone in oil every 3 days to vaginal progesterone TID. A previous randomized controlled study did find a benefit to 50 mg PIO IM every third day added to 200 mg vaginal progesterone BID compared to 200 mg vaginal progesterone BID alone[10]. This data and that of others suggest that variability in PGT-A genetic testing laboratory does impact ongoing pregnancy rates [11]. We saw a range of fetal heartbeat per euploid embryo from 44 to 58% depending on the genetic testing laboratory. Differences in outcomes by genetic testing laboratory may be due to interpretation algorithms or methodology. The LifeView platform developed and used by Genomic Prediction and licensed to Ovation uses a high-density SNP platform while the other labs use NGS.

In conclusion, FET protocol and the PGT-A genetic testing laboratory had associations with the ongoing pregnancy rate after single euploid FETs at our center. Although there was no significant association of embryologist that performed the embryo biopsy or the physician that performed the transfer and the ongoing pregnancy rates, these may be impactful variables at other centers. These data suggest that modified natural cycles may be the best FET protocol. Letrozole probably decreases the success rates of modified natural FETs. Each clinic should be familiar with their pregnancy rates using HRT FETs compared to modified natural FETs without using oral medication for ovarian stimulation.

## Data Availability

A limited data set is available through Mendeley Data.
